# Magnetic Nanoparticles Enhance Pore Blockage-Based Electrochemical Detection of a Wound Biomarker

**DOI:** 10.3389/fchem.2019.00438

**Published:** 2019-06-12

**Authors:** Gayathri Rajeev, Allison J. Cowin, Nicolas H. Voelcker, Beatriz Prieto Simon

**Affiliations:** ^1^Future Industries Institute, University of South Australia, Mawson Lakes, SA, Australia; ^2^Faculty of Science, Institute for Biomedical Materials and Devices, University of Technology, Sydney, NSW, Australia; ^3^Drug Delivery, Disposition and Dynamics, Monash Institute of Pharmaceutical Sciences, Monash University, Parkville, VIC, Australia; ^4^Melbourne Centre for Nanofabrication, Victorian Node of the Australian National Fabrication Facility, Clayton, VIC, Australia; ^5^Commonwealth Scientific and Industrial Research Organisation, Clayton, VIC, Australia; ^6^Department of Materials Science and Engineering, Monash University, Clayton, VIC, Australia

**Keywords:** magnetic nanoparticles, porous anodic alumina membrane, pore blockage, electrochemical biosensor, chronic wound, nanoporous materials

## Abstract

A novel pore blockage-based electrochemical immunosensor based on the combination of 100 nm-magnetic nanoparticles (MNPs), as signal enhancers, and 200 nm-pore diameter nanoporous anodic alumina (NAA) membranes, as sensing platform, is reported. A peptide conjugate mimicking flightless I (Flii), a wound healing biomarker, was chosen as target analyte. The sensing platform consists of an anti-Flii antibody (Ab1)-modified NAA membrane attached onto a gold electrode. Anti-KLH antibody (Ab2)-modified MNPs (MNP-Ab2) were used to selectively capture the Flii peptide conjugate in solution. Sensing was based on pore blockage of the Ab1-modified NAA membrane caused upon specific binding of the MNP-Ab2-analyte complex. The degree of pore blockage, and thus the concentration of the Flii peptide conjugate in the sample, was measured as a reduction in the oxidation current of a redox species ([Fe(CN)_6_]^4−^) added in solution. We demonstrated that pore blockage is drastically enhanced by applying an external magnetic field at the membrane backside to facilitate access of the MNP-Ab2-analyte complex into the pores, and thus ensure its availability to bind to the Ab1-modified NAA membrane. Combining the pore blockage-based electrochemical magnetoimmunosensor with an externally applied magnetic field, a limit of detection (LOD) of 0.5 ng/ml of Flii peptide conjugate was achieved, while sensing in the absence of magnetic field could only attain a LOD of 1.2 μg/ml. The developed sensing strategy is envisaged as a powerful solution for the ultra-sensitive detection of an analyte of interest present in a complex matrix.

## Introduction

There is a growing demand for rapid, highly sensitive, simple-to-fabricate, and cost-effective biosensing platforms for the detection of biological targets in medical diagnostics. Conventional methods like enzyme-linked immunosorbent assays (ELISAs) are not desirable as diagnostic platforms in clinical setups as they require long analysis time, sample preparation and several incubation and rinsing steps prior to analysis.

Electrochemical biosensors translate the presence of analytes into electrical signals (Grieshaber et al., 2008) and are suitable to develop point-of-care (POC) handheld sensing devices since electrodes can be easily miniaturized by methods such as screen printing (Yamanaka et al., 2016), photolithography (Mir et al., 2010) or ink-jet printing (Silveira et al., 2016) which allow easy integration into electronic devices (Zhu et al., 2014).

With substantial advancements in nanotechnology, remarkable improvement in the sensing performance of electrochemical biosensors has been achieved using nanoparticle (NP)-based signal amplification (Zhu et al., 2014). Nowadays, different kinds of functional nanomaterials such as metal NPs, quantum dots, nanoporous materials, carbon-based nanomaterials, and magnetic NPs (MNPs) have been used to design ultra-sensitive biosensing platforms (Lei and Ju, 2012; Walcarius et al., 2013; Holzinger et al., 2014).

MNPs have been extensively used in magnetic separation of biomolecules from complex biological samples and as solid supports for performing immunoassays (Rocha-Santos, 2014). Advances in biosensing strategies that employ magnetic labels have resulted in platforms that push the limits of detection to very low levels resulting in devices that can be used for early disease detection in medical diagnostics (Dittmer et al., 2008). Nowadays, a variety of commercial MNPs are readily available with a wide range of sizes, magnetic properties, and surface functionalities that are used in biosensors. They have been used for capturing analytes from complex samples, leaving behind undesired molecules in the background matrix, and labeling analytes (Van Reenen et al., 2014).

The main advantages of using MNPs include their large surface area, ease of functionalization, lack of harmful effects to the human body, controllable size, and simple manipulation in magnetically actuated devices. Surface functionalities on MNPs allow their surface modification with biorecognition elements that feature high affinity to the analyte of interest, whereas the large surface-to-volume ratio increases the number of bioreceptors available to react with the analyte, contributing to increase the sensitivity of the device (Dittmer et al., 2008; Gijs et al., 2009; Peyman et al., 2009). The latter effect can be further enhanced by the feasibility of MNPs to facilitate pre-concentration of analytes prior to analysis, being key for applications that require ultra-sensitivity to detect minute concentrations of analyte. Additionally, biofunctionalized MNPs can isolate specific analytes of interest from complex biological samples by magnetic actuation, which can minimize matrix effects thereby reducing the occurrence of false positives.

Lab-on-chip technologies that integrate multiple analytical functions in a single chip are of great interest to scientists due to the growing demand for easy-to-use diagnostic devices (Huang and Mason, 2013). Such devices enable the end users to perform all the steps from sampling to obtaining easily readable results in a single platform. MNPs have been used in developing such bioassays (Rocha-Santos, 2014; Van Reenen et al., 2014). MNPs allow integration into microfluidic devices where they can be manipulated within the microfluidic channels by applying an external magnetic force (Giouroudi and Keplinger, 2013). This can lead to the design of miniaturized technologies based on MNPs which significantly reduce sample volume and analysis time.

After specific analyte capture using MNPs, a detection step is crucial to provide accurate and specific detection of the analyte-MNP complex. Several strategies have been explored by researchers for the detection of the analyte-MNP complexes (Gijs et al., 2009). Labeling strategies are commonly used, where the captured analyte is labeled with a fluorescent dye (Peyman et al., 2008, 2009; Tarn et al., 2010; Sasso et al., 2012), a chemiluminescent molecule or an enzyme, that enable further detection. Thorough washing or separation steps are required when using labeled strategies so that only the captured analytes are labeled and only the bound labels are detected. Using fluorescent molecules as labels results in high limits of detection (LODs) since the background fluorescence from the MNPs is significant and thus the fluorescence from the label itself is comparatively weak (Peyman et al., 2009). Chemiluminescent molecules are also commonly used as labels and are considered one of the most sensitive immunoassays where the intensity of luminescence emitted from chemical reactions is measured (Zhang et al., 2011; Kim and Lim, 2015). Although they exhibit very low LODs (Wang et al., 2012), chemiluminescent emission intensities are sensitive to environmental factors such as temperature, solvent ionic strength, pH, and other species present in the system. Moreover, emission intensity from the chemiluminescent reaction varies with time. Therefore, the emission versus time profile varies greatly from one compound to another (Baeyens et al., 1998). When enzymes are used as labels in enzyme-linked immunosorbent assays (ELISAs), addition of substrates that convert the enzymes into a measurable signal is required (Sista et al., 2008). Enzymatic labeling has the advantage that the signal is amplified by the enzymatic conversion process. However, several incubation and washing steps are required, which are not desirable. Alternatively, the use of MNPs as labels in surface binding assays has been explored where the analyte-MNP complexes bind specifically on a surface in a sandwich format (Morozov and Morozova, 2006; Dittmer et al., 2008). To this purpose, specific bioreceptors with affinity toward two different epitopes of the analyte are required to be immobilized on the MNPs and on the surface.

Nanoporous anodic alumina (NAA) membranes have been demonstrated as suitable substrates for developing biosensing platforms owing to their exceptional features such as highly ordered nanopores, tunable pore geometry, high surface-to-volume ratio, thermal and mechanical stability, biocompatibility, and chemical resistance (Jani et al., 2013; Santos et al., 2013; Rajeev et al., 2018a). Various electrochemical biosensors have been developed using NAA as the sensing platform (Santos et al., 2013; Rajeev et al., 2018b). NAA membranes functionalized with specific bioreceptors have been used to electrochemically detect various analytes such as DNA (De La Escosura-Muñiz and Merkoçi, 2010), proteins (De La Escosura-Muñiz and Merkoçi, 2010), and pathogens (Cheng et al., 2012; Nguyen et al., 2012). Detection is carried out by monitoring changes either in impedance or current intensity upon specific analyte binding to the immobilized bioreceptor, which partially blocks the diffusion of a redox species through the nanopores toward the underlying electrode surface. De la Escosura-Muñiz et al. demonstrated the use of NAA membrane for *in situ* monitoring of parathyroid hormone-like hormone (PTHLH) secretion in cultured human cells using electrochemical detection (De La Escosura-Muñiz et al., 2018). Recently, De la Escosura-Muñiz et al. also developed a methodology for electrical monitoring of virulence factors secreted by bacterial pathogens using NAA membranes (De La Escosura-Muñiz et al., 2019). AuNP tags have been used by Merkoçi's group as pore blockage agents in NAA-based electrochemical biosensors. Signal enhancement using AuNPs in a sandwich immunoassay approach aimed to detect an IgG lowered the LOD to 50 ng/ml compared to a LOD of 500 μg/ml achieved by an equivalent NAA immunosensor based on direct detection (De La Escosura-Muñiz and Merkoçi, 2010, 2011).

In this work, we report a pore blockage-based electrochemical magnetoimmunosensor where the MNPs are demonstrated as pore blockage enhancers. Results show that simply applying a magnetic force to pull the analyte-MNP complex toward the sensor surface significantly pushes the LOD down, being 2,400-folds lower than that achieved in the absence of magnetic field. The combination of NAA pore blockage-based sensing systems along with the unique features of MNPs in immunosensing is highly promising to develop diagnostic platforms fulfilling the requirements of point-of-care devices. There have been other studies published in the past using gold or magnetic nanoparticles (MNP) as pore blockage enhancers on NAA-membranes. For example, in a work published by Ye et al. (2016), NAA membranes integrated into a PDMS chamber with a Pt electrode as working electrode were combined with the use of MNP as pore blockage enhancers for histamine detection in seafood. In another work published by De La Escosura-Muñiz and Merkoçi (2011), they used a NAA membrane on a screen-printed carbon electrode as sensor surface and AuNPs as pore blockage enhancers for the detection of proteins in whole blood. In this work, for the first time we demonstrated the use of magnetic field to concentrate MNP bound analytes on the sensor surface to enhance the pore blockage.

Flightless I is a negative regulator of wound healing which is present at high level in chronic, non-healing wounds (Ruzehaji et al., 2012). Flightless neutralizing antibodies (FnAb) have been developed by Cowin group. Use of these neutralizing antibodies show that Flii can be depleted from wound fluid and that they have a positive therapeutic effect on chronic wounds (Cowin et al., 2007; Kopecki et al., 2012; Ruzehaji et al., 2014). Detection of Flii proteins in chronic wounds is of great interest in chronic wound research since it may be an indication of the wound status in non-healing wounds. However, wound fluid is a complex biological sample containing a plethora of biomolecules involved in wound healing which can cause significant matrix effects during biosensing. MNPs can help to tackle this issue, as they are known to isolate specific biomolecules from complex biological samples, eliminating or at least reducing potential interferences.

We aim here to develop a pore blockage-based biosensing platform with sensitivity enhancement via MNP-boosted pore blockage as a proof of concept for the electrochemical detection of Flii proteins. Isolated or recombinant whole Flii protein is not readily available. Hence, a synthetic peptide corresponding to the active region of Flii protein conjugated to a keyhole limpet hemocyanin (KLH) carrier protein is used in this study as a model analyte that facilitates a sandwich immunoassay. While FnAbs are used as immobilized antibodies on the NAA sensor surface, commercial polyclonal anti-KLH antibodies are used as the bioreceptors on the MNP surface. Anti-KLH antibodies displayed on the MNPs specifically capture the KLH-Flii peptide conjugate from solution. MNPs with bound analyte are then incubated onto the FnAb-modified NAA sensor surface to perform a sandwich assay within the nanopores. Partial blockage of the pores upon binding impedes free diffusion of a redox species, [Fe(CN)_6_]^4−^, toward the underlying gold transducer surface, measured as a decrease in its oxidation current intensity. We compared the sensing performance in the presence and absence of magnetic field to study the effect of the magnetic attraction on facilitating the access of the KLH-Flii peptide conjugate-MNP immunocomplex into the pores and thus on the achieved sensitivity.

## Materials and Methods

### Reagents

NAA membranes of 200 nm pore diameter (Whatman Anodisc Circles 13 mm, 200 nm) were purchased from Interpath services (Australia). Carboxyl-terminated MNPs (100 nm diameter, 25 mg/ml, fluidMAG-ARA) were purchased from Chemicell (Germany). Potassium ferrocyanide (K_4_[Fe(CN)_6_]), potassium ferricyanide (K_3_[Fe(CN)_6_]), *N*-hydroxysuccinimide (NHS), *N*-(3-dimethylaminopropyl) *N*′-ethylcarbodiimide hydrochloride (EDC), ethanolamine, phosphate buffered saline (PBS) tablets, 3-(triethoxysilyl) propyl isocyanate (ICN) silane, 3,3′,5,5′-tetramethylbenzidine (TMB) substrate, and 2-(N-morpholino)-ethanesulfonic acid (MES) were purchased from Sigma-Aldrich (Australia).

Polyclonal antibodies against KLH produced in rabbit and anti-human IgG antibodies (used as control) were purchased from Sapphire Bioscience (NBP1-30443, Australia). Monoclonal antibodies raised in mouse against the N-terminus of the LRR domain of the human Flii protein (FnAb) were developed and supplied by Cowin group (Jackson et al., 2012). Custom made KLH-Flii peptide conjugate was purchased from Mimotopes (Australia).

### Preparation and Characterization of Antibody-Modified MNPs (MNP-Ab2)

Carboxyl-terminated MNPs were modified with anti-KLH antibodies (Ab2) via carbodiimide chemistry. One microliter of MNPs, which is equivalent to 4.5·10^10^ particles, was added to 100 μl of MES buffer, pH 5. MNPs were washed by 5 min-incubation in MES buffer with good mixing in an orbital shaker, followed by 4 min-capture on a magnet to allow supernatant removal. This washing step was performed 3 times. Carboxyl groups on the washed MNPs were activated to form NHS ester groups by reacting with a 1:1 mixture of 100 μl of 0.4 M of EDC and 0.1 M of NHS in cold MES buffer. This reaction was conducted under good mixing in an orbital shaker for 30 min at room temperature. Then, the activated MNPs were captured on a magnet for 5 min and the supernatant was removed. Activated MNPs were incubated in 100 μl of 50 μg/ml of Ab2 in 0.01 M PBS, pH 7.6, for 1 h at room temperature. Antibody-modified MNPs were washed 4 times in PBS containing 0.01% Tween 20, following the washing protocol previously described, to remove any non-specifically bound antibodies. Ab2-modified MNPs (MNP-Ab2) were incubated in 100 μl of 0.1 M ethanolamine in PBS for 1 h to block any remaining active ester groups on the MNPs surface. Modified MNPs were stored at 4°C until further use.

Fourier transform infrared (FTIR) spectroscopy was conducted after each surface modification step. FTIR spectra were obtained using a Vertex 70 Hyperion microscope Bruker in reflectance mode (Bruker Optics, Germany) over the range of 650–1,000 cm^−1^ at a resolution of 4 cm^−1^ and averaging 64 scans. Background spectra were taken on a gold-coated glass slide. Samples for FTIR characterization were prepared by placing a small drop of unmodified MNP solution, NHS ester-activated MNP solution, and Ab-modified MNP solution on the surface of the gold slide and drying them in a desiccator under vacuum to generate a thin layer of MNPs. Sample spectra were recorded and data analyzed using the OPUS 7.2 spectroscopy software (Bruker).

### Preparation and Characterization of the NAA Immunosensing Surface

Isocyanate groups were introduced on the surface of the NAA membranes with 200 nm pores via silanization to facilitate further covalent immobilization of the bioreceptor FnAb. Briefly, NAA membranes were boiled in 30% H_2_O_2_ for 1 h to obtain fresh hydroxyl groups and remove any organic contaminants. Hydroxylated membranes were dried under a mild nitrogen flow and baked in an oven at 60°C for 2 h to remove any remaining water content. This is a key step since the silanes are highly reactive to water. Then, the membranes with active hydroxyl groups were immersed in a 5% solution of ICN silane in dry toluene in a sealed reaction vessel under nitrogen atmosphere. Silanization was carried out for 2 h under constant shaking at room temperature and inert atmosphere, which resulted in isocyanate groups (-N = C = O) displayed on the NAA surface. After silanization, the functionalized membranes were washed with fresh dry toluene and dried under a nitrogen stream. Finally, they were incubated with a 50 μg/ml FnAb (Ab1) solution in 0.1 M PBS (pH 7.4) for 2 h under constant shaking. After incubation, membranes were washed thoroughly with copious amount of PBS, and stored in PBS at 4°C until they were used for sensing experiments.

FTIR spectroscopy was used to characterize the membranes surface after each step of modification. FTIR spectra were obtained using the method described in section Preparation and Characterization of Antibody-Modified MNPs (MNP-Ab2).

Scanning electron microscopy (SEM) was used to characterize the surface morphology of the NAA membranes.

### Analyte (KLH-Flii-Conjugate) Isolation Using MNP-Ab2

**Scheme 1:**
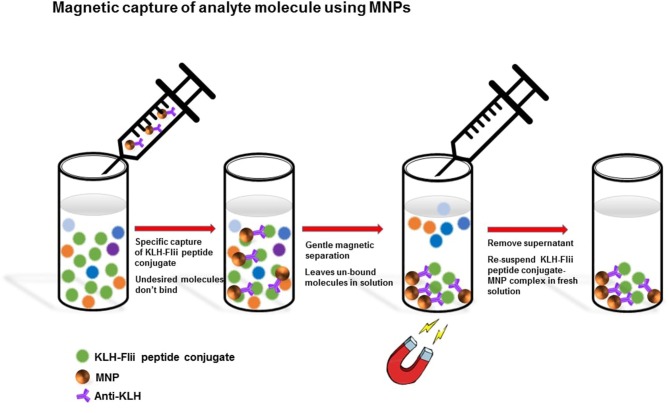
Isolation of KLH-Flii peptide conjugate using MNPs.

MNPs-Ab2 were used to capture the KLH-Flii peptide conjugate from solution as shown in Scheme 1. MNPs-Ab2 were incubated in 100 μl of Flii peptide conjugate at various concentrations (2.5, 5, 10, 20, and 40 μg/ml) in PBS for 1 h at room temperature with good mixing. Afterwards, MNPs-Ab2 with captured analyte (MNP-Ab2-Flii) were washed 3 times in PBS solution containing 0.01% Tween 20 using the protocol previously described and the washed MNP-Ab2-Flii complex was re-suspended in fresh PBS and used for sensing experiments.

### ELISA to Detect MNP-Ab2-Flii Complex

Detection of MNP-Ab2-Flii complex was initially carried out by colorimetric ELISA to confirm the feasibility to use the involved immunospecies in a sandwich assay and to compare the sensing performance of this colorimetric assay with that achieved by the novel NAA-based electrochemical immunosensor which uses MNPs as pore blockage enhancers. Briefly, isolated MNP-Ab2-Flii complex was incubated first in 100 μl of 50 μg/ml Ab1 in PBS for 1 h followed by incubation in a 1:5,000 dilution of HRP-labeled anti-mouse IgG in PBS. Every incubation step was followed by 3 washing steps in PBS containing 0.01% Tween 20 followed by one washing step in PBS. One hundred μl of TMB were added as HRP substrate. The catalytic conversion of the chromogenic substrate TMB into a colored product by the enzyme used as label was followed by measuring the absorbance at 650 nm, allowing target quantification.

### Electrochemical Detection of MNP-Ab2-Flii Complex on a NAA-Based Immunosensor

Electrochemical sensing was conducted using an electrochemical analyser (CH Instruments, model 600 D series, USA) and a three-electrode system in a Teflon cell. Ab1-modified NAA membrane mounted on a gold-coated glass slide (Au/NAA-Ab1) acted as the working electrode, a silver/silver chloride electrode (CH Instruments, USA) was the reference electrode and a platinum wire was the counter electrode. Electrochemical measurements were done using a 2 mM K_4_[Fe(CN)_6_] and 2 mM K_3_[Fe(CN)_6_] solution in 0.1 M PBS. Differential Pulse Voltammetry (DPV) was used as detection technique in which a series of regular voltage pulses were applied ranging from −0.3 to 0.8 V and the oxidation current of the redox species was measured at 0.18 V.

One hundred μl of MNP-Ab2-Flii complex in PBS, obtained upon incubation of MNP-Ab2 in solutions of different concentrations of Flii peptide conjugate (2.5, 5, 10, 20, and 40 μg/ml), were incubated on the immunosensor surface for 1 h at room temperature. DPV measurements were performed before and after incubating the MNP-Ab2-Flii complex solutions. Each experiment was performed in triplicate. Control experiments were done using an anti-human IgG-modified NAA-based sensor to evaluate whether the MNP-Ab2-Flii complex was non-specifically adsorbed on the membrane.

### Sensitivity Enhancement Using External Magnetic Field

A strong external magnetic field using a large rare earth magnet made from neodymium, iron, and boron-NdFeB (Jaycar Electronics, Australia) was applied at the backside of the immunosensing surface to study its effect on the sensing performance by providing an additional force to attract the MNP-Ab2-Flii complex into the pores and thus facilitates its binding to the Ab1 immobilized on the NAA surface. We hypothesize that this novel approach can enhance MNP-Ab2-Flii complex binding by overcoming diffusion limitations that limit analytes entering the pores, and thus amplify the sensing signal and eventually enhance the sensitivity. In the absence of magnetic field, the MNP-Ab2-Flii complex enters the pores only by diffusion and gravitational forces. The externally applied magnetic field acts as an extra force to attract the MNP-Ab2-Flii complex which facilitates its effective path toward the sensor surface and thus its exposure and final binding to the immobilized Ab1, thereby acting as pore blockage enhancers. Stringent washing while placing the magnet on top of the sensor was required after the MNP-Ab2-Flii complex incubation under magnetic field to remove any MNPs that may be non-specifically bound or trapped within the pores.

## Results and Discussion

### Immobilization of Anti-KLH on MNPs and FnAb on NAA Membranes

**Figure 1:**
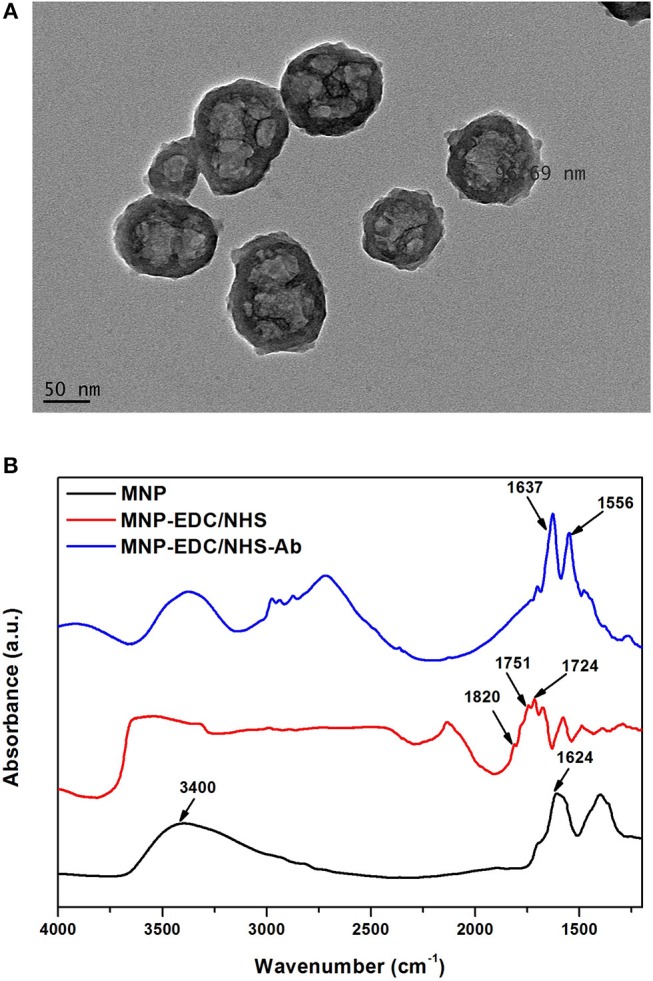
TEM characterization of MNPs **(A)**, and FTIR spectra of MNPs at different modification steps: unmodified MNPs (black) after EDC/NHS activation (red) and after Ab2 immobilization (blue) **(B)**.

MNPs were used to harvest KLH-Flii peptide conjugate from solution. To that purpose, first anti-KLH antibody (Ab2) was covalently bound to carboxyl-terminated MNPs. Carboxyl-terminated MNPs have a particle size of 100 nm, confirmed using TEM (Figure 1A). Carbodiimide chemistry is the most versatile method to activate carboxylic groups for their binding to primary amines, EDC being the most popular carbodiimide used for this purpose. NHS was used in the EDC coupling protocols to improve the efficiency of the reaction by creating an amine-reactive intermediate. EDC couples NHS to carboxyl groups and forms an NHS ester which is more stable than the O-acylisourea intermediate formed in the absence of NHS. The NHS ester intermediate reacts with the amine groups of the antibody to form stable amide bonds. Figure 1B presents the FTIR spectra at different modification steps of MNPs. The spectrum of the carboxyl-terminated MNPs before activation (black) shows a strong band at 1,624 cm^−1^ and a broad band at 3,400 cm^−1^ which are characteristic of C = O stretching and O-H stretching, respectively. This confirms the presence of carboxylic groups on the MNPs surface. EDC/NHS activation results in an NHS ester intermediate, reflected in the corresponding FTIR spectrum (red) as a characteristic triplet peak at 1,724, 1,751, and 1,820 cm^−1^ (Böcking et al., 2008; Sam et al., 2009). The bands at 1,724 and 1,751 cm^−1^ correspond to the C = O anti-symmetric and C = O symmetric stretching vibrational modes of the succinimidyl ring, respectively. The band at 1,820 cm^−1^ corresponds to the C = O symmetric stretching vibrational mode and the C = O stretching vibrational mode of the succinimidyl ester. After antibody binding, the FTIR spectrum (blue) shows two bands at 1,556 and 1,637 cm^−1^ that confirm the formation of amide bonds between the activated MNPs and the antibody. These bands correspond to the amine I and amine II vibrations, respectively.

**Figure 2:**
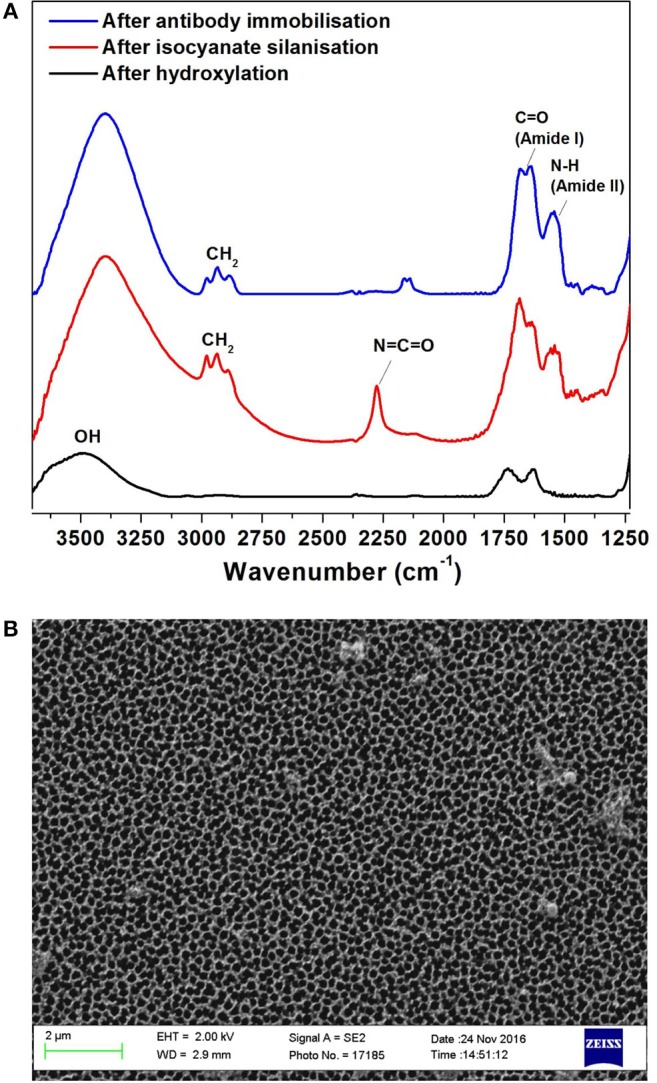
FTIR spectra of NAA immunosensing surface at different modification steps: after hydroxylation (black), silanization (red) and Ab1 immobilization (blue) **(A)**, and SEM top-view image of NAA **(B)**.

The surface for immunosensing was prepared as detailed in the experimental section Preparation and Characterization of the NAA Immunosensing Surface. Silanization chemistry was used to immobilize FnAb within the porous layer. The NAA porous surface is characterized by a high density of native hydroxyl groups. These groups were activated by boiling in H_2_O_2_ at 70°C for 1 h. This step also removes any organic contaminants from the surface and forms fresh hydroxyl groups. The hydroxylated surface was functionalized by means of silanization chemistry using ICN silane. This produced a dense monolayer of ICN silane with highly reactive isocyanate groups (N = C = O) on the NAA surface. This group readily reacted with the amine groups of FnAb. FTIR spectroscopy was used in reflectance mode to confirm the surface modification steps of the NAA membranes. Figure 2A presents the FTIR spectra at different NAA surface modification steps. The broad peak at 3,340 cm^−1^ in the black spectrum in Figure 2A corresponds to hydroxyl groups on NAA surface after hydroxylation. The spectrum of the NAA surface after reacting the hydroxylated membrane with ICN silane (red spectrum in Figure 2A) showed a characteristic band at 2,250 cm^−1^ representing N = C = O stretching vibration mode, confirming the successful silanization and presence of isocyanate groups on the surface. Bands at 2,925 and 2,854 cm^−1^ were assigned to the stretching vibration modes of the aliphatic CH_2_ groups in the silane (Dronov et al., 2011). In this same spectrum, bands at 1,550 and 1,650 cm^−1^ were present which are characteristics of C = O stretching and N-H bending vibration modes. The presence of these bands is attributed to the fast hydrolysis undergone by some of the isocyanate groups upon reaction with residual water in the solvent or water vapor in the atmosphere. The blue spectrum in Figure 2A was obtained after covalent immobilization of FnAb on the silanized NAA surface. It confirms successful immobilization of antibodies via the disappearance of the isocyanate band at 2,250 cm^−1^ and the presence of intense bands at 1,550 cm^−1^ (N-H bending vibration mode) and 1,650 cm^−1^ (C = O stretching vibration mode) from the peptide bonds formed between the antibodies and the isocyanate groups (Dronov et al., 2011).

Scanning SEM was used to characterize the surface morphology of the NAA membrane (Figure 2B). The top view of the membranes revealed that they had an average pore diameter of 207 ± 11 nm.

### Detection of MNP-Ab2-Flii Complex on NAA Membranes

**Figure 3:**
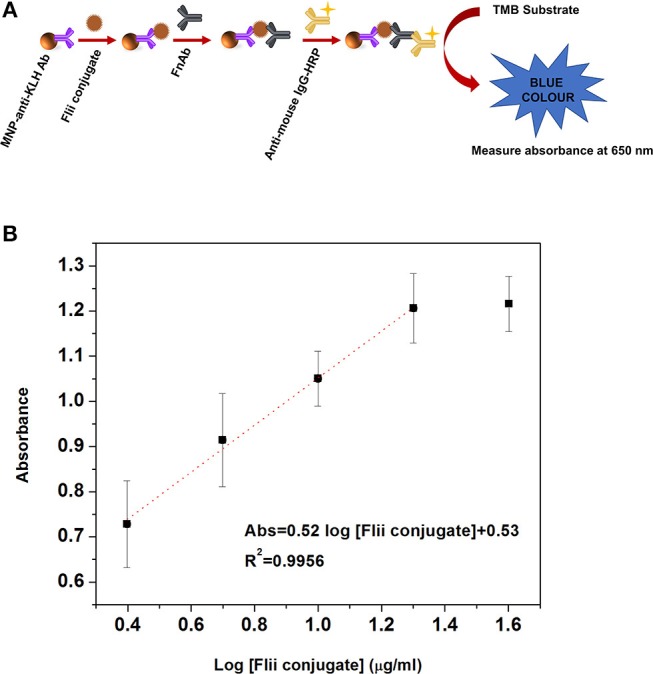
Scheme **(A)** and dose response curve **(B)** for the colorimetric ELISA detection of Flii peptide conjugate, previously isolated using MNPs.

Before conducting electrochemical sensing experiments on the NAA immunosensing surface, initially we performed a colorimetric ELISA as described in the experimental section ELISA to Detect MNP-Ab2-Flii Complex, to confirm that the sandwich format works well and to compare its sensing performance with that of the NAA-based immunosensor. Figure 3A shows the scheme for the sandwich colorimetric ELISA. Absorbance was measured at 650 nm and plotted against the logarithm of KLH-Flii peptide conjugate concentrations (2.5, 5, 10, 20, and 40 μg/ml). The colorimetric ELISA detection based on a sandwich assay provided a LOD of 2.9 μg/ml, calculated using the equation 3Sa/b, where Sa is the standard deviation of the y-axis and b is the slope of the linear range of the calibration curve (Figure 3B). The sensitivity of this system was found to be 0.52 ml/μg.

The sensitivity achieved by the described ELISA is expected to be improved by the developed NAA-based electrochemical immunosensor combining the properties of MNPs, NAA membrane and pore blockage-based electrochemical transduction method as explained previously.

**Scheme 2:**
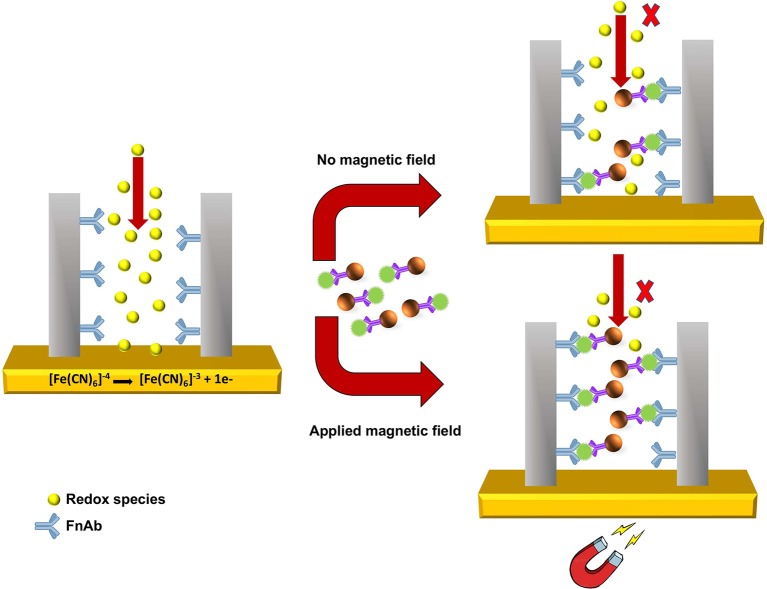
Sensing principle of pore blockage-based electrochemical detection using MNPs as enhancers.

As shown in Scheme 2, pore blockage was used as sensing principle to detect MNP-Ab2-Flii complex on a NAA-based immunosensor. Pore size is key to allow access of the immunocomplexes and maximize blockage, and thus sensitivity, upon binding to the immobilized antibodies. Formation of sandwich immunocomplexes within the pores partially blocks the diffusion of the redox species present in solution toward the gold transducer surface. This results in a reduction in the voltammetric oxidation signal of [Fe(CN)_6_]^4−^ to [Fe(CN)_6_]^3−^ used to quantify the concentration of KLH-Flii peptide conjugate.

The pore blockage sensing principle using NAA membranes has been demonstrated as an efficient label-free biomolecule detection approach (De La Escosura-Muñiz and Merkoçi, 2010; De La Escosura-Muñiz et al., 2013; Espinoza-Castañeda et al., 2015). We show here the advantages of using MNPs in combination with a pore blockage-based immunosensor, not only to enhance the sensitivity, but also to capture the analyte from complex samples and thus avoid matrix effects caused by interfering compounds. Additionally, manipulation of MNPs by an external magnetic field can be utilized to facilitate the access of MNPs containing the immunocomplex into the pores to increase their availability for immunoreaction with the antibodies immobilized on the pores surface. This amplification strategy is expected to significantly improve the sensing sensitivity. Hence in this research, we demonstrate the use of MNPs as pore blockage enhancers and the possibility to further increase the sensitivity by applying an external magnetic field.

**Figure 4:**
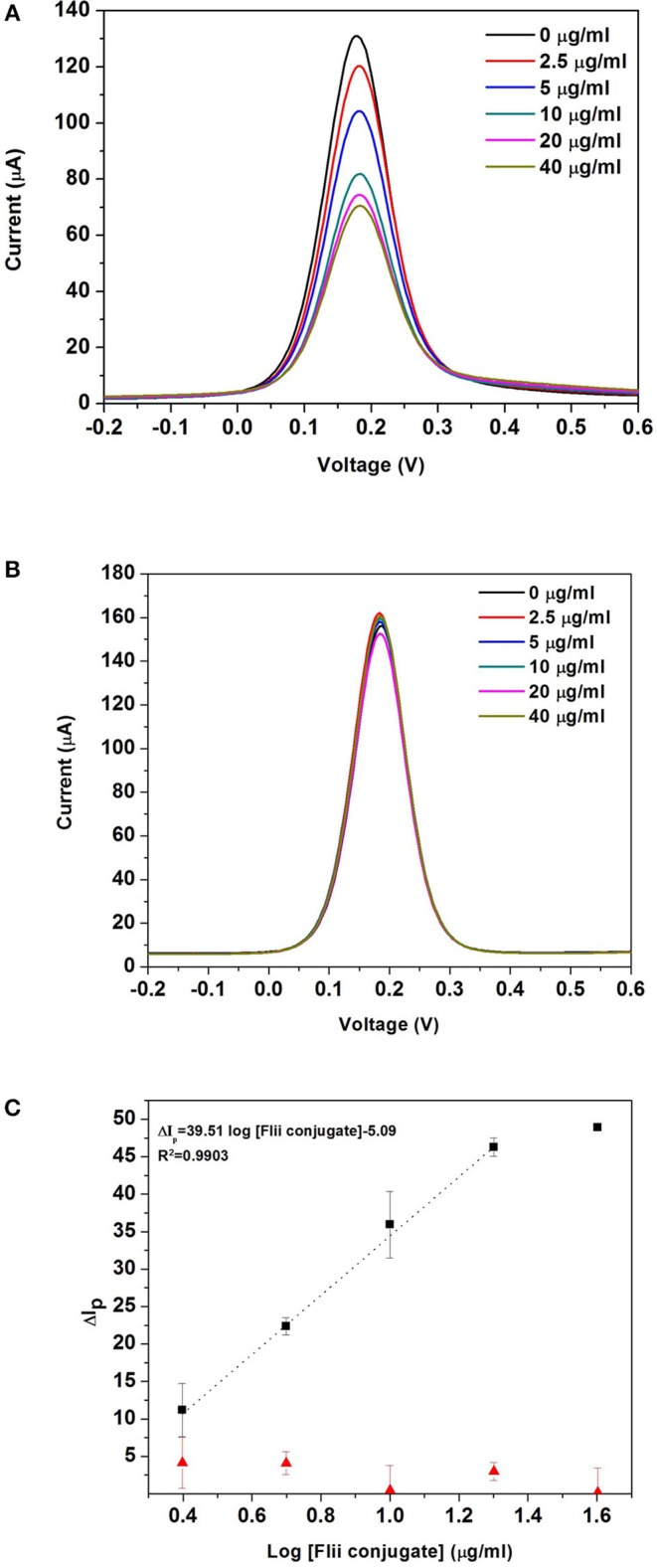
DPV curves obtained with the NAA-based electrochemical immunosensor **(A)**, and the control sensor **(B)** in the absence of magnetic field; dose response curves for the detection of KLH-Flii peptide conjugate plotted using the NAA-based electrochemical immunosensor and control sensor in the absence of magnetic field **(C)**. Error bars are calculated as standard deviation from the average of three individual experiments.

Figure 4A shows the DPV curves obtained after incubating the MNP-Ab2-Flii complex solutions, obtained upon incubation of MNP-Ab2 in various solutions with KLH-Flii peptide concentrations ranging from 2.5 to 40 μg/ml, on the NAA-based immunosensor in the absence of magnetic field. As expected, the oxidation current decreased with binding of the MNP-Flii complex obtained upon incubation with increasing concentrations of KLH-Flii peptide conjugate. To prove the specific binding between MNP-Flii peptide conjugate complex and the FnAb immobilized on the pores surface, a control experiment was performed using a NAA-based sensor modified with an anti-human IgG (Figure 4B).

To evaluate the sensing performance, current intensity changes were normalized using the following equation:

$$\begin{array}{l} {\text{I}}_{\text{p}}=\frac{\left({\text{I}}_{\text{0}}\text{-}{\text{I}}_{\text{p}}\right)}{{\text{I}}_{0}}\;\times 100 \end{array}$$

where Δ*I*_p_ was the % current intensity change and *I*_0_ and *I*_p_ were the current intensity values measured prior and after incubation with MNP-Ab2-Flii peptide conjugate. Calibration curves were obtained by plotting Δ*I*_p_ as a function of log [Flii peptide conjugate] (maximum standard deviation of 4.4%; Figure 4C). The response from the control sensor (red triangles in Figure 4C) was negligible compared to the response from the Flii NAA immunosensor (black squares in Figure 4C), proving that the sensing response was due to specific binding between the MNP-Flii peptide conjugate complex and the Ab1 immobilized on the pores surface. The LOD was found to be 1.2 μg/ml, being only somewhat better than that obtained by ELISA (Figure 3B) which is 2.9 μg/ml. However, the sensitivity of the proposed sensing system was 76 times higher (40 ml/μg) than that of the ELISA which had a sensitivity of 0.52 ml/μg. The proposed NAA-based electrochemical immunosensor significantly improved the performance of the ELISA even in the absence of magnetic field. Reproducibility of the developed immunosensors was calculated as the relative standard deviation of the sensitivity of three individual sensors, being 3.8%. Encouraged by the outstanding improvement in sensitivity of the NAA-based immunosensor using MNPs as pore blockage enhancers, we then combined the same detection strategy with the application of an external magnetic field. We expect this approach to further improve the sensitivity by facilitating access of the immunocomplex into the pores, as described in the next section.

### Sensitivity Enhancement Using External Magnetic Field

Our initial hypothesis was that the sensing performance of the developed pore blockage-based electrochemical immunosensor could be greatly enhanced in terms of sensitivity by applying an external magnetic field at the backside of the electrode. In the presence of the magnetic field, MNPs with bound analyte molecules are actuated in the direction of the magnetic field. This is an additional force for the MNPs to go through the pores apart from diffusion and gravitational pull when sensing is performed in the absence of magnetic field. This facilitates the binding of the MNP-Ab2-Flii complex to the bioreceptors (FnAb) immobilized on the pores surface and thus enhances the pore blockage effect. The immunosensor was placed on top of a large rare earth magnet made from neodymium, iron and boron-NdFeB during MNP-Ab2-Flii complex incubation. After that, thorough washing was performed with the magnet on top of the pores to magnetically drive any unbound MNPs stuck inside the pores outside the membrane by placing the magnet on top of the sensor.

**Figure 5:**
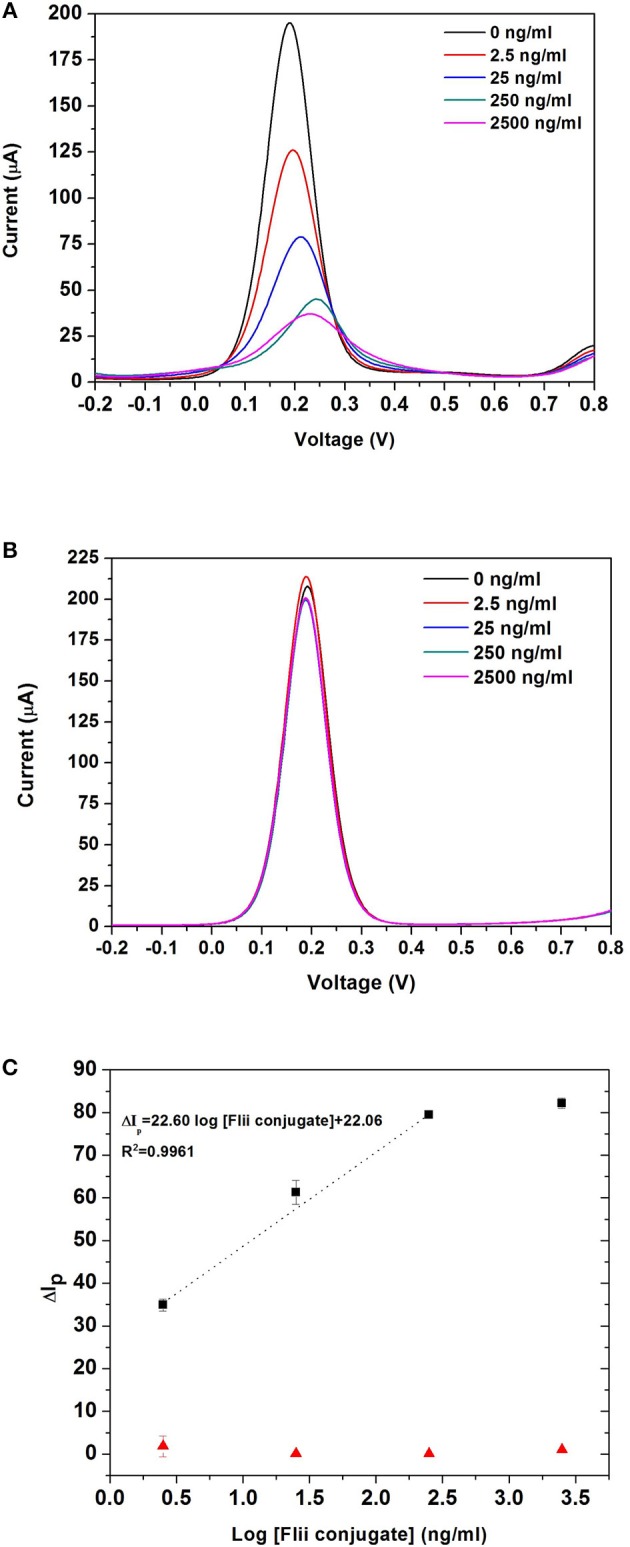
DPV curves obtained with the NAA-based electrochemical immunosensor **(A)**, and the control sensor **(B)** when an external magnetic field is applied during the analyte incubation step; dose response curves for the detection of KLH-Flii peptide conjugate plotted using the NAA-based electrochemical immunosensor and control sensor when an external magnetic field is applied during the analyte incubation step **(C)**. Error bars are calculated as standard deviation from the average of three individual experiments.

Sensing experiments were performed by incubating MNP-Ab2-Flii complex solutions obtained upon MNP-Ab2 incubation in various KLH-Flii peptide conjugate solutions with concentrations ranging from 2.5 ng/mL to 2.5 μg/mL. DPV curves before and after incubation of MNP-Ab2-Flii complex on a NAA-based immunosensor and a control sensor, in the presence of an external magnetic force, are shown in Figures 5A,B, respectively. Calibration curves were plotted as explained previously to evaluate the sensing performance (maximum standard deviation of 2.8%; Figure 5C). The response from the control sensor (red triangles in Figure 5C) was negligible compared to that from the specific NAA immunosensor (black squares in Figure 5C). A LOD of 0.5 ng/mL was obtained, providing 5,800 and 2,400-fold enhancement, respectively, compared to the results obtained using ELISA and the same NAA-based immunosensor in the absence of magnetic field. A sensitivity of 27·10^3^ml/ μg was achieved which is 673 times higher than that obtained by the immunosensor in the absence of magnetic field. Application of an external magnetic field to manipulate the particles seems to enhance the pore blockage effect as demonstrated by the ability to detect KLH-Flii peptide conjugate at concentrations 1,000-folds' lower than in the absence of magnetic field. The immunosensor performance in terms of sensitivity and LOD was greatly enhanced using the developed sensing strategy by magnetically manipulating the access of MNPs into the pores, while still showing an excellent reproducibility among sensors (2.8%).

These results confirm that by combining the use of MNPs as pore blockage agents and the application of an external magnetic field during analyte incubation, the NAA-based electrochemical immunosensor performance can be greatly improved when pore blockage is used as sensing mechanism. This sensing system has the potential to be extended to detect low concentrations of small molecules. Due to the ability of MNPs to isolate a target analyte from a complex biological sample, it may well be suitable to detecting biomarkers in samples with complex matrices.

## Conclusions

We report here the development of a pore blockage-based biosensing platform with sensitivity enhancement via MNPs as a proof of concept for the electrochemical detection of Flii proteins. We have successfully demonstrated the proof-of-principle of the use of MNPs as signal enhancers in combination with FnAb-modified NAA platforms based on pore blockage as sensing mechanism. A synthetic peptide corresponding to the active region of the wound biomarker Flii protein conjugated to a KLH carrier protein was used as a model analyte. MNPs were modified with an anti-KLH antibody to specifically capture the Flii-KLH conjugate. Then, the MNP-Ab2-Flii complex was subsequently captured by the FnAb antibodies within the NAA porous structure causing partial pore blockage. Pore blockage was used as sensing principle, being measured as a reduction in the voltammetric oxidation current of a redox species added in solution. Enhancement in pore blockage was achieved by applying an external magnetic field which pulled the MNP-Ab2-Flii complex more efficiently toward the sensor. This resulted in a significant enhancement in the analytical signal achieving a LOD of 0.5 ng/ml, while the LOD achieved in the absence of magnetic field was 1.2 μg/ml. Our results confirm that this novel sensing strategy can be used for developing biosensing platforms with enhanced sensing performance.

## Author Contributions

The experiments presented in this work were designed by GR, AC, NV, and BP. GR conducted the experimental work. GR, NV, and BP discussed the results obtained from the experiments. GR wrote the manuscript and the last version was revised by all authors (GR, AC, NV, and BP). All authors read and approved the final manuscript.

### Conflict of Interest Statement

The authors declare that the research was conducted in the absence of any commercial or financial relationships that could be construed as a potential conflict of interest. The handling editor is currently organizing a Research Topic with one of the authors, NV, and confirms the absence of any other collaboration.
